# Effects of artificial canopy shading on vegetative growth and ripening processes of cv. Nero d’Avola (*Vitis vinifera* L.)

**DOI:** 10.3389/fpls.2023.1210574

**Published:** 2023-09-15

**Authors:** Daniele Miccichè, Maria Inès de Rosas, Massimo Vincenzo Ferro, Rosario Di Lorenzo, Stefano Puccio, Antonino Pisciotta

**Affiliations:** ^1^ Department of Agricultural, Food and Forest Sciences, University of Palermo, Palermo, Italy; ^2^ Universidad Nacional de Cuyo, Facultad de Ciencias Agrarias, Laboratorio de Fisiología Vegetal, Mendoza, Argentina

**Keywords:** shading, grapevine, berry composition, *Vitis vinifera*, berry development, climate change, light, temperature

## Abstract

**Introduction:**

The biology of the grapevine (*Vitis vinifera* L.) is clearly influenced by the climatic conditions of the growing environment, where temperature and light play a major role in modifying plant physiology. In the scenario of climatic changes, radiative excess, correlated to the increase in temperature, can concretely subject the photosynthetic apparatus to a condition of light saturation and cause a drastic reduction in photochemical efficiency, giving rise to chronic photoinhibition phenomena. Undoubtedly, the ripening behavior also undergo evident alterations; the problem of sugar ripening, which is often strongly accelerated, is induced not only by high temperatures but also by the excess concentration of carbon dioxide (CO_2_), which results in a higher ripening. In addition, high berry temperatures favor a reduction in the concentration of organic acids. The reported trends indicate that the need for urgent action is closely linked to the future environmental impacts that these changes could have on the entire wine sector. In recent years, shade treatments have been applied to the vine canopy to overcome this issue.

**Methods:**

The objective of this study was to determine how artificial canopy shading affects the vines vegetative growth and the ripening processes of *Vitis vinifera* cv. Nero d’Avola during the 2019-2020 vegetative seasons. Three treatments were established: shading treatment with a green net (shade factor 27%), shading treatment with a white net (shade factor 32%), and untreated vines, thus naturally exposed to light radiation.

**Results and discussion:**

Artificial shading, applied at full fruit set, interfered with the microclimate of the vines, causing partial effects on the grape ripening processes. At harvest, significant differences were found between the treatments in terms of sugars, also shading treatments increased must acidity and decrease pH. Results obtained on vegetative parameters, suggest that the shading treatment delays leaf fall, with potentially positive effects on the starch accumulation on perennial reserve organs to be exploited at the following season’s sprouting. Shading significantly reduced berry size, with obvious consequences on bunch weight and yield per vine. In 2020, shaded plants showed a delay in all the phenological phases. The total anthocyanins content was not changed by the shading treatment. The results obtained confirm the importance of net coverage on the microclimate of the vines, vegetative-productive activity, and grapes quality. From this point of view, the net covering technique can be a tool for controlling grapes ripening dynamics in the context of climate change.

## 1 Introduction

Since the 1950s, substantial changes in climate have occurred, according to the Intergovernmental Panel on Climate Change (IPCC, 2022). These changes concern not only temperatures exceeding seasonal averages, but also an intensification of extreme events such torrential rainfall alternated with long drought periods (Easterling et al., 2000).

Grapevine development is highly sensitivity to variations in the thermal regimes (Van Leeuwen and Darriet, 2016). Viticultural regions are conventionally situated between 30° to 50° N and 30° to 40° S (Amerine et al., 1980). Hence thermal increase might shift this vineyard regions towards higher latitudes (Jones et al., 2005a; Jones et al., 2005b; Moriondo et al., 2013). Considering this, non-conventional vineyard management as shading could be a way to preserve traditional agricultural locations.

The Mediterranean region has historically been ideal for growing vines, and the wine produced there is highly prized and consumed all over the world. Nero d’Avola, with approximately 15.5 hectares, is the second most cultivated variety in Sicily and accounts for just 16% of the area under wine grapes in the entire region. It is considered a ubiquitous cutltivar since it is present in each of Sicily’s nine provinces and, moreover, in five of them it is still the most representative variety. Nero d’Avola accounts for 50-70% of the wines of Sicily’s only denomination of controlled and guaranteed origin (DOCG). At national level, it is suitable for cultivation in regions of central and southern Italy such as Tuscany, Latium, Apulia and Calabria. (AAVV, 2018).

Unfortunately, the Mediterranean Region has been pointed as a hotspot area of climate change damage map causing the reduction in its suitability for grapevine cultivation (Mozell and Thach, 2014; Ashenfelter and Storchmann, 2016; Santillán et al., 2020). Projections for this area show a decrease in precipitation of between 10% and 40%, and a temperature increase of 1°C to 3.7°C by the end of the century (IPCC, 2015; Mariotti et al., 2015; Caloiero et al., 2018; Todaro et al., 2022). The predicted loss of suitability has already begun and it is evident in events such as earlier phenology (Malheiro et al., 2013; de Cortázar-Atauri et al., 2017; Bernáth et al., 2021) and its consequences on wine quality (Fraga and Santos, 2017). However, the early ripening and thus early harvest date, seems to be a cultivar dependent issue (Biasi et al., 2019). Early phenology triggers premature ripening to happen during the period of higher temperatures and thus, a “thermal decoupling” phenomenon occurs, causing a delay in phenolic accumulation, but not in sugar increase during berry maturation (Sadras and Moran, 2012; Arrizabalaga et al., 2018). To address this problem, most winegrowers tend to delay the harvest date in favor of a more adequate phenolic ripening; however, this choice results in negative effects on the composition of the grapes, which develop high sugar contents and high pH, as well as lower acidity (Lobos et al., 2015; Lu et al., 2021). As a result, particularly alcoholic wines are produced, which are not appreciated by the modern consumer (Palliotti et al., 2014).

The described scenario suggests the need of defining clear short-term adaptation strategies to climate change and shading may be useful tool to undergo this issue. Previous studies have attempted to understand the effects of bunch shading using boxes in Shiraz (Downey et al., 2004). Results suggested that the accumulation of flavonoids, such as anthocyanins, is not strictly light dependent. Indeed, it is also linked to the temperature levels reached by the bunches, as it has been shown for cv. Merlot (Spayd et al., 2002). In cv Shiraz the shading treatment has a partial influence on technological ripening, as pH and titratable acidity were higher, but total soluble solids (TSS) were the same for both, covered and uncovered treatments (Ristic et al., 2007). On the other hand, Scafidi et al. (2013) reported that shading the bunches of Grillo with propylene boxes resulted in a lower accumulation of sugars, proanthocyanidins and aromas.

However, more recent studies have investigated the effect of shading not only the bunches, but the entire canopy using shade nets in cvs in Semillon, Shiraz and Cabernet Sauvignon (Greer et al., 2011; Greer and Weedon, 2012; Caravia et al., 2016; Lu et al., 2021). The results suggest that canopy shading, when applied from veraison (BBCH 81) to harvest, has clear effects on ripening delay, evidenced by lower TSS accumulation (Caravia et al., 2016) and increased titratable acidity (Greer et al., 2011; Greer and Weedon, 2012). These effects can be traced back to interference from the shade net with the photosynthetic processes of the canopy. The supply of sugars to clusters is reduced as the photosynthetic efficiency of the leaves decreases due to shading. Conceptually, similar results might be obtained by green vine pruning, implemented with the aim of causing a delay in ripening processes (Palliotti et al., 2013).

Overall, these evidence show that the main effects of shading are related to the shift on vine microclimate environment (mostly temperature, humidity and PAR light) and as a consequence, to the changes on phenology, anthocyanins, pH, acidity and TSS, so far. However, the consequences of shading seem to vary in a cultivar and environment-dependent manner, leading to the need of understanding the best cultural practice for the Mediterranean vineyard region and for Nero d’Avola, being the most important cultivar of the area. In addition, by shading the entire canopy, and not just the bunches, a better understanding of the vine physiology can be accomplished and allows to manage the entire plant throughout vintages. Still, a main issue remains unsolved: the adequate percentage of shading (covering net characteristics) to accomplish the required quality requirements for wine grapes. The differences in shading percentage may produce diverse vine microclimates and may reach the cooling effect desired to ameliorate the climate change detrimental effect on grapes quality (Cugnetto and Masoero, 2021). This work studies over two vintages the effect of different types of covering net, in terms percentage of shading, on grape quality, shoot fertility, and reserve replenishment on cv Nero d’Avola, the most important grape variety for this region. The combined effect of PAR, temperature and humidity produced by the nets on grapevine physiology leads eventually to different microclimates. This microclimate management could be a useful tool to mitigate climate change detrimental effects on berry (and wine) quality, according to the characteristics/variation of each season, with minimum handling, within a sustainability framework.

## 2 Materials and methods

### 2.1 Experimental site and plant material

The trial was conducted in a commercial Nero d’Avola/140 Ru (*Vitis vinifera* L.) vineyard, located in Western Sicily, Italy, (37°39’04,2” N, 12°59’41,8” E; 360 m a.s.l.), over two consecutive seasons (2019 – 2020). The vines were planted in 2005. The rows were oriented NW-SE and spaced 1.00 m in rows and 2.40 m between the rows. Vines were VSP trained, and Guyot pruned (8 buds along the cane and 2 buds along the spur). The vineyard was not irrigated. The climate was semi-arid according to Köppen-Geiger classification (Falquina et al., 2022) and the soil presented a prevalent clay texture. The vines were trellised in a vertical shoot-positioned system and, cane-pruned (Guyot system: 8 and 2 buds per cane and spur, respectively). The cane was set at 0.85 m above ground with two pairs of catchwires providing trellising 0.7 m above the canes. During the study, double shoots were pruned at the end of May, before the flowering. Conventional cultivation practices for the production of healthy grapes were used mainly addressed to Oidium (Erysiphe necator) and Peronospora (Plasmopara viticola).

### 2.2 Experimental design and treatments

Shade treatments (ST) were applied from pea-size until harvest (BBCH 73-89), during the 2019 and 2020 vegetative seasons as follows: (i) a white net (WN) 1 m wide, 32% shade factor, made of Arlene HT polyethylene UV stabilized, and a green net (GN) 1 m wide, 27% shade factor, made of 100% HD polyethylene UV stabilized (Arrigoni SPA, Italy), both used for the partial canopy shade treatment (ST). Untreated control (UC) received no shade treatment and was compared to the two shading treatments. The shade net covered 1 m of the canopy at about 0.8 m from the soil level. Three replicates of each treatment were arranged in randomized blocks designed and distributed in three adjacent rows. Each replicate included a plot of 30 vines with similar growth vigor. Evaluations were carried out on the 15 middle vines of each row, leaving the other vines as borders among the treatments. During the two-seasons, the nets were permanently installed but applied only between pea-size (BBCH71) and harvest.

### 2.3 Macro and microclimates variables

During the two years of this trial, weather data were collected through an automated weather station at the Servizio Informativo Agrometeorologico Siciliano, 2022 (www.sias.regione.sicilia.it) located less than ten km to the vineyard. Measurements of temperature (°C), relative humidity (%), and PAR (µmol/m^2^/s) were made in each treatment to characterize the microclimatic modifications induced by the shading treatments. Canopy climatic parameters (temperature and relative humidity) were monitored at bunch level every 60 minutes using one WatchDog sensor (1000 Series, Spectrum Technologies, Bridgend, UK) per treatment from BBCH 71 to BBCH 89. In both seasons, photosynthetically active radiation (PAR: wavelength range 400 - 700 nm) (µmol/m^2^/s), captured inside the canopy, was measured by a ceptometer (Apogee LQS 70-10M, Apogee Instruments, Logan, UT), positioned at the height of the fruiting zone and parallel to the row direction. Measurements were done during six different phenological stages (BBCH 71 – 73 – 77 – 81 – 85 – 89), in both exposures, at three specific time intervals (10:00-11:00 am; 12:00-1:00 pm; 3:00-4:00 pm), and at five randomly selected points in each of the three replicates per treatment.

### 2.4 Vegetative parameters, vigor, and plant nutritional status

In the 2020 season ten and thirty days after bud break, the phenological stage of the three treatments was determined according to Eichorn and Lorenz (1984). This monitoring was done by evaluating all cane buds on a sample of 15 plants per treatment. At harvest, 15 shoots per treatment were randomly selected from 15 vines and used to determine the length and leaf area per shoo throughLI-3100C area meter (Li-COR Environmental, 4647 Superior Street Lincoln, NE, USA). Leaf area per plant was calculated as the product of shoot leaf area times the number of plant shoots. The nutritional status of the plants was evaluated non-destructively by measuring the following parameters immediately after full veraison: leaf chlorophyll (CHL) and flavonoid (FLAV) content, nitrogen balance index (NBI - ratio of CHL to FLAV), foliar anthocyanin (ANT) content using the Dualex 4 scientific optical leaf clip meter (Dx4, FORCE-A, Orsay, France). The instrument determines epidermal absorbance in UV-A (wavelength 315-400 nm), mainly due to flavonoids, by comparing chlorophyll fluorescence signals at two different excitation wavelengths (375 and 650 nm) (Goulas et al., 2004; Cartelat et al., 2005). Measurements were made on 15 shoots per treatment; shoot selection was done by identifying 5 proximal, 5 middle, and 5 distal shoots of the cane; for each shoot, three leaves from the basal, median and apical position of the shoot were identified. Measurements were performed at the beginning of veraison (BBCH 81) and at full ripening process (BBCH 89) on the central part of the leaf avoiding the main veins. During winter dormancy, pruning weight was determined by collecting and weighing pruned material from 15 vines per experimental unit. Ravaz’s index (yield/pruning weight) was then calculated (Ravaz and Sicard, 1903).

### 2.5 Yield and berry characteristics – must quality

During each season, all treatments were harvested at the same time, when the UC vines had reached 23.5° Brix, a level considered suitable for the required quality. At harvest, the number of bunches per vine, the weight of the bunches and the yield per vine were determined in 15 vines per treatment. Berry weight was determined measuring the weight of individual berries on a sample of 300 berries per treatment from veraison (BBCH 81) to full ripening (BBCH 89). On the grape juice coming from the pressing of 0.5 kg sample of berries, were determined the Total Soluble Solids Content (TSS) (°Brix) using a digital refractometer (model HI96811, Hanna instruments, Padova, Italy). Titratable Acidity (TA) (g/L) and pH were determined by acid/base titration and pH-meter (model HI99111 Hanna instruments, Padova, Italy). Sugar loading expressed in mg per berry, was calculated based on berry fresh mass and sugar concentration (Deloire, 2011).

### 2.6 Statistical analysis

Significant differences in microclimate, fruit quality and physiological parameters were analyzed by two-way ANOVA, comparing treatments and seasons, Tukey test for p<0.05. (Minitab 17 Statistical Software, 2010, State College, PA: Minitab, Inc.). Diurnal temperature was compared to PAR using a linear regression. The coefficient of determination (R^2^) was considered significant for p ≤ 0.001.

## 3 Results

### 3.1 Vineyard macroclimate

**Figure 1:**
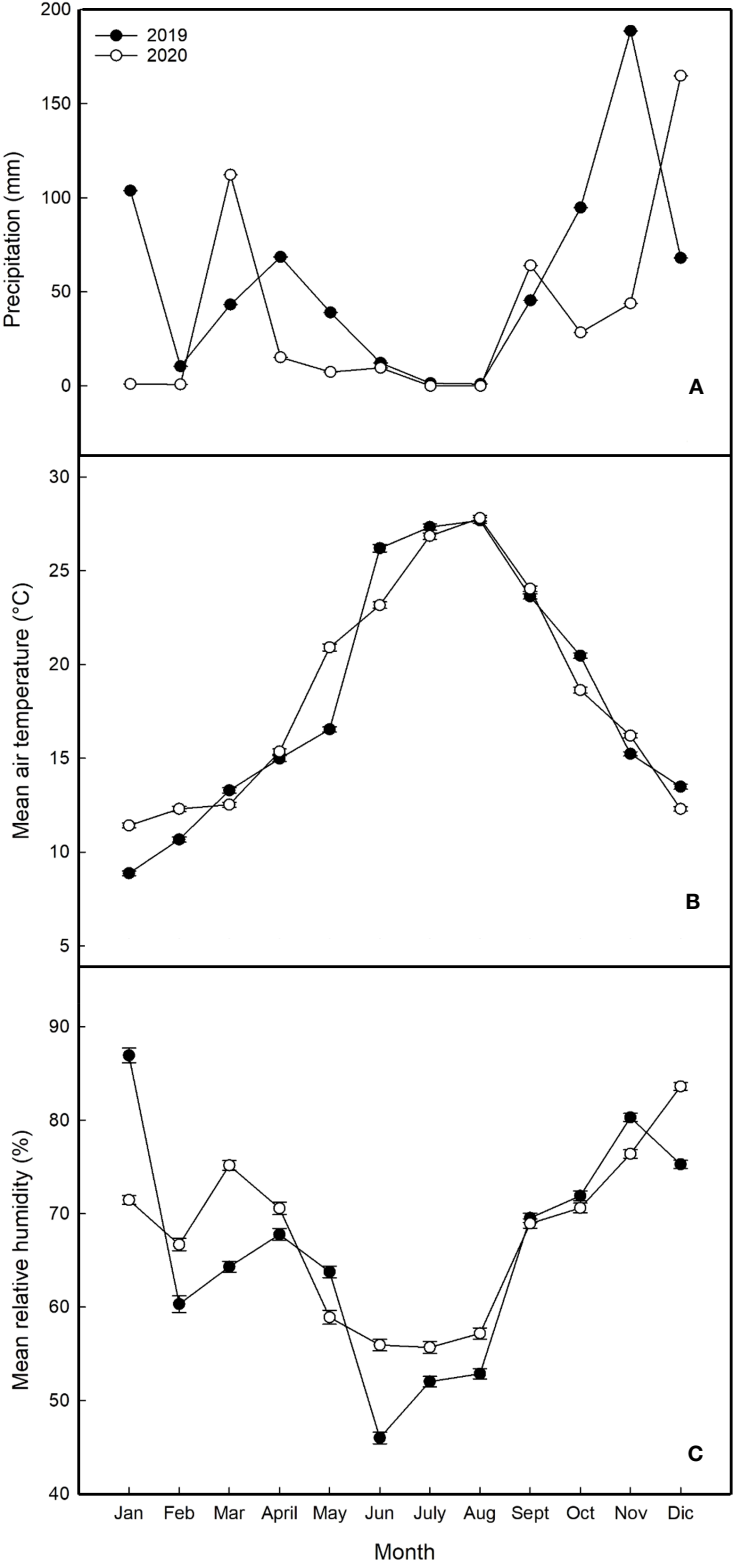
Seasonal climatic trends at the experimental site during the years 2019 and 2020 **(A)** average monthly precipitation; **(B)** average monthly air temperatures; **(C)** average monthly relative humidity).

Climatic data obtained from the regional station showed that 2020 was drier and warmer than 2019, but higher humidity was observed during summertime. Total precipitation was 676 mm in 2019 and 446mm in 2020 (34% less) (**Figure 1A**). In both years, rainfall was concentrated during winter months, 61% and 72% in 2019 and 2020 respectively. The temperatures were higher in 2020 mostly during winter months. The average temperature in January and February was 28% and 15% higher than these two months in 2019, respectively (**Figure 1B**). As far as spring, May was also warmer in 2020 (26.4%) when compared to 2019. During summer, both years were mostly similar regarding temperatures. In concern to relative humidity, mostly higher levels of it were observed from February to August in 2020 when compared to 2019 (**Figure 1C**).

### 3.2 Microclimatic condition

The two-way ANOVA test shown a significant effect on photosynthetically active radiation (PAR) only by the ‘treatment’ factor. The shading treatments (WN and GN) reduced the photosynthetically active radiation (PAR) compared to control vines (UC) in the three-time slots evaluated (p= 0.000). The attenuation of intercepted radiation varied according to the technical characteristics of the nets (hole size, and shading factor) and the time of day. In the time slot 10.00-11.00, a reduction in intercepted radiation of 12.7% and 17.6% were observed for GN and WN, respectively, compared to UC. In the next time interval (12.00-13.00), although ambient light radiation increased by about 340 µmol/m^2^/s, the attenuation of the intercepted radiation showed a similar trend (**Table 1**). Likewise, the ST response was the same between 15.00-16.00 hs, when the lowest PAR values were intercepted (14.3% and 18.2% less than UC, in GN and WN, respectively). The ‘year’ factor and the ‘YxT’ interaction were not statistically different for PAR radiation measurements.

**Table 1:** Photosynthetically active radiation (PAR) was intercepted by the two shading treatments (under the net) and control in three-time slots.

Treatment	PAR (µmol m^-2^ s^-1^)		External PAR	PAR %
10.00 a.m. - 11.00 a.m.				
UC	727.3 ± 13.1	a	1249.3	58.2
WN	507.5 ± 8.0	b	1249.3	40.6
GN	568.3 ± 9.0	b	1249.3	45.5
12.00 p.m. - 1.00 p.m.				
UC	964.1 ± 15.6	a	1591.9	60.6
WN	697.8 ± 12.4	b	1591.9	43.8
GN	753.0 ± 12.9	b	1591.9	47.3
3.00 p.m. - 4.00 p.m.				
UC	675.5 ± 20.0	a	1147.3	58.9
WN	467.1 ± 17.2	b	1147.3	40.7
GN	511.2 ± 17.4	b	1147.3	44.6

WN, white net; GN, green net; UC, control. Different letters within a column represent significant differences (Tukey’s test, p-value <0.05), n.s. = not significant. Values are reported as means ± SE.

**Figure 2:**
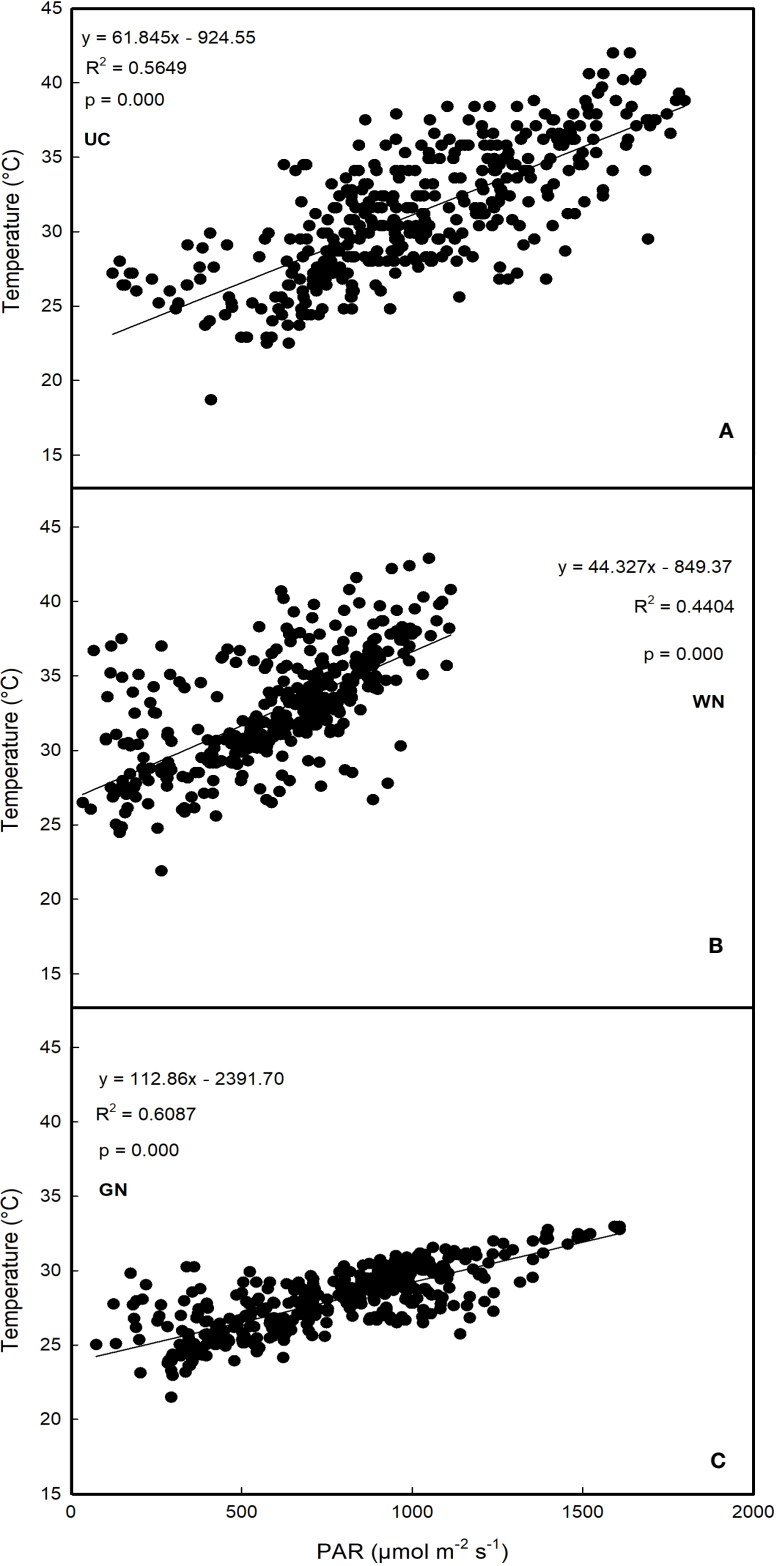
Regression analysis between photosynthetically active radiation and average daytime temperature **(A)** untreated control; **(B)** white net; **(C)** green net.

Further considerations regarding PAR concern its relationship with mean diurnal temperatures for each treatment (**Figure 2**). The results show that GN resulted in the highest correlation values (R^2^ = 0.6087), followed by UC (R^2^ = 0.5649). The lowest correlation level was recorded for WN (R^2^ = 0.4404).

**Figure 3:**
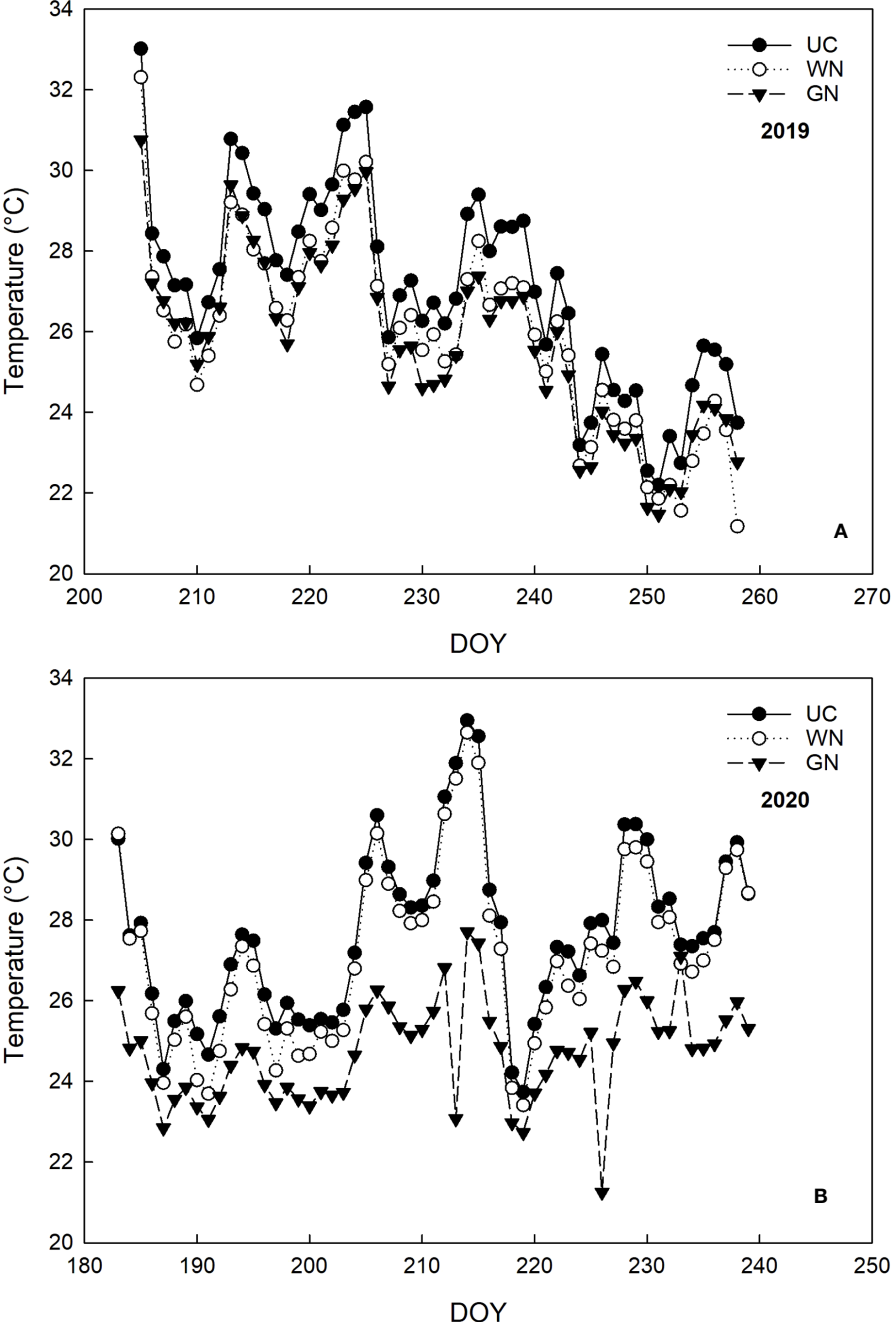
Monitoring the temperature (° C) of the three treatments during the years 2019 **(A)** and 2020 **(B)**.

The air temperature on the vines’ environment varied according to the treatments and the vintages (“YxT”, (p=0.000)). Regarding the 2019 vintage from the covering time (BBCH 71) until the veraison (BBCH 81), GN and WN significantly reduced the mean temperature by 6.8% and 5.5%, respectively, compared to UC (**Figure 3A**). During ripening (BBCH 81- BBCH 89), the mean temperatures of GN and WN were about 2°C lower than UC. Moreover, the number of hours in which temperatures were above 32° C were 220 for UC, corresponding to an increase of about 100% and 44% compared to GN and WN, respectively (**Table 2**). In 2020, after the application of ST, until the fruit set, the average daytime temperature decreased between 4.9% and 12.2% compared to UC (**Figure 3B**). In the later stages of the season, the differences between GN and UC were maintained, as the latter showing 12% higher average temperature values. In contrast to this and to 2019, the differences between UC and WN were less evident. Further analysis supports these results, as both the number of hours with temperatures above 32° C and the average temperature above 32° C are higher in UC (**Table 2**). Monitoring of night-time temperatures showed no effect of ST compared with UC. The two-way ANOVA test showed that the RH parameter was not significant for the shade treatment factor nor “YxT”. On the contrary, as might be expected for different seasons variations, there was an effect of the “year” factor (RH, p < 0.001).

**Table 2:** Monitoring of temperature and number of hours above 32° C of the three theses during the 2019 and 2020 seasons.

Variable	Year	UC		WN		GN	
∑ h 30-32° C	2019	72.0		55.0		55.0	
	2020	73.0		67.0		109.0	
∑ h >32° C	2019	220.0		152.0		111.0	
	2020	302.0		261.0		13.0	
T mean 30-32° C	2019	31.0 ± 0.3		31.1 ± 0.3	n.s.	31.1 ± 0,3	
	2020	30.9 ± 0.3	b	31.3 ± 0.3	a	30.7 ± 0,3	c
T mean >32° C	2019	36.1± 0.7	a	34.7 ± 0.5	b	34.1 ± 0,3	c
	2020	36.0 ± 0.6	a	35.7 ± 0.4	a	32.5 ± 0,3	b

The data were subjected to one-way analysis of variance (ANOVA) (Tukey’s test, p-value <0.05) and different letters within a row indicate a statistically significant difference and n.s. = not significant. Values are reported as means ± SE.

### 3.3 Phenology, vine growth, and nutritional status

#### 3.3.1 Phenology stages

**Figure 4:**
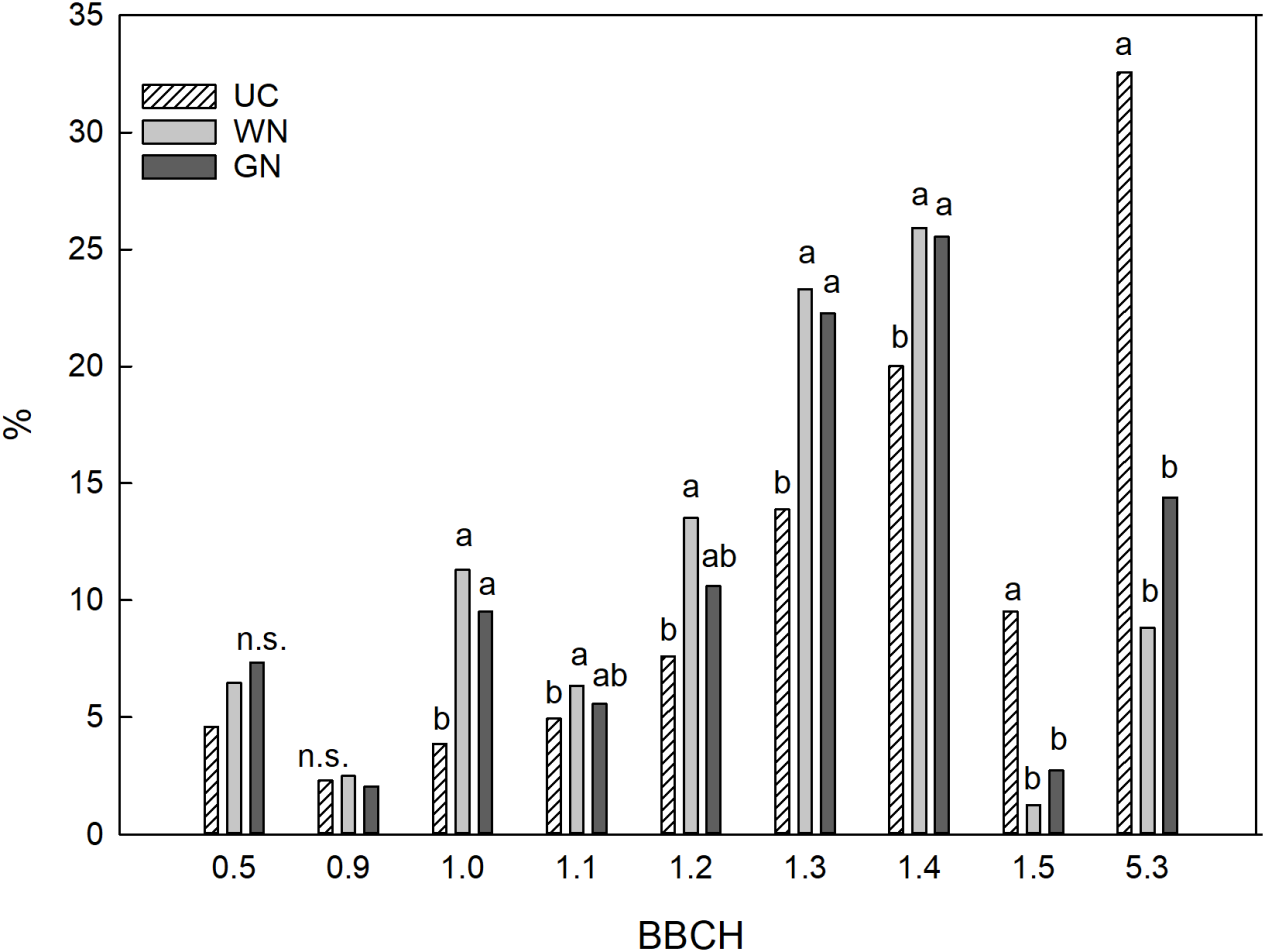
Phenological stages of buds thirty days after bud burst. The data were subjected to one-way ANOVA (Tukey’s test, p-value <0.05), and different letters indicate a statistically significant difference.

The shading treatments delayed the phenological development of the vines. The phenological stages from bud break to flowering were the same among the treatments at the beginning of the study (10 days after bud break). 30 days after bud break, the UC vines were in an advanced stage of development in comparison to those in both shaded treatments (**Figure 4**). It was observed that 32.6% of the control buds had reached the ‘clearly visible inflorescence’ stage (BBCH 53), while only 14.4% and 8.8% of GN and WN vines had done so, respectively. Additionally, more ST buds were observed at the leaf development stage of three or four open leaves (BBCH 13 - BBCH 14) in comparison to UC vines.

#### 3.3.2 Shoot length, leaf area, and pruning weight

The shoot length was higher in UC in both years (“YxT”, p = 0.001) (**Table 3**); however, the number of leaves at harvest was higher in ST than in UC (“YxT”, p = 0.001). Specifically, the number of leaves per shoot at harvest in the WN treatment was 35% and 34.4% higher (compared to UC) in 2019 and 2020, respectively, while in the GN treatment, it was 46.7% and 23.3% higher correspondingly. This result shows that vines grown under shaded conditions have lower leaf drop, resulting in higher leaf area values at harvest (“YxT”, p = 0.001). Consequently, in 2019 there was an increase in leaf area per vine compared to UC of between 13.3% (WN) and 20% (GN). For The following season, ST resulted in an average increase of 29% compared to UC. Analysis of values for Ravaz’s index showed no significant difference (data not shown).

**Table 3:** Vegetative parameters of the untreated control and shade treatments measured at harvest (BBCH 89).

Variable	Treatment	2019		2020	
SL (cm)	UC	131.5 ± 6.2	a	175.2 ± 6.1	a
	WN	113.3 ± 7.9	b	162.1 ± 5.8	b
	GN	107.9 ± 7.6	b	154.3 ± 7.3	b
LS (n)	UC	12.0 ± 0.9	b	18.9 ± 1.4	b
	WN	16.2 ± 1.4	a	23.3 ± 1.0	a
	GN	17.6 ± 1.4	a	25.4 ± 1.7	a
LA_1_(m^2^)	UC	0.12 ± 0.05	b	0.19 ± 0.06	b
	WN	0.17 ± 0.03	a	0.26 ± 0.08	a
	GN	0.14 ± 0.04	a	0.25 ± 0.05	a
LA_2_(m^2^)	UC	1.51 ± 0.06	b	1.39 ± 0.07	b
	WN	1.72 ± 0.15	a	1.84 ± 0.06	a
	GN	1.84 ± 0.16	a	1.81 ± 0.14	a

SL, Shoot length; LS, leaf per shoot; LA1, primary shoot leaf area; LA2, leaf area per vine. Different letters within a column represent significant differences (Tukey’s test, p-value <0.05), n.s. = not significant. Values are reported as means ± SE.

#### 3.3.3 Nutritional status

Two-way ANOVA analysis of ecophysiological parameters reports a “YxT” effect only for flavonoids (p=0.023). CHL and ANTH attributes were significant for both the “year” and “treatment” factors, individually. In the case of NBI, only an effect of the “treatment” factor was shown.

In 2019, the shading influenced the flavonoid content of leaves; specifically, for UC, direct exposure of leaves to light radiation led to an increase in this parameter by 9.3% and 2.8% compared to WN and GN, respectively. This trend was also observed during the second season; in 2020, control vines showed significantly higher flavonoid content than ST, with increases ranging from 15% (GN) to 23% (WN) (**Table 4**).

**Table 4:** Flavonoids (Flav) content in leaves of the untreated control (UC) and the two shade treatments (WN-GN).

Variable	Treatment	2019		2020	
Flav (µg/cm^2^)	UC	1.30 ± 0.03	a	1.44 ± 0.03	a
	WN	1.19 ± 0.04	b	1.16 ± 0.03	b
	GN	1.26 ± 0.02	ab	1.25 ± 0.04	b

Different letters within a column represent significant differences (Tukey’s test, p-value <0.05), n.s. = not significant. Values are reported as means ± SE.

**Figure 5:**
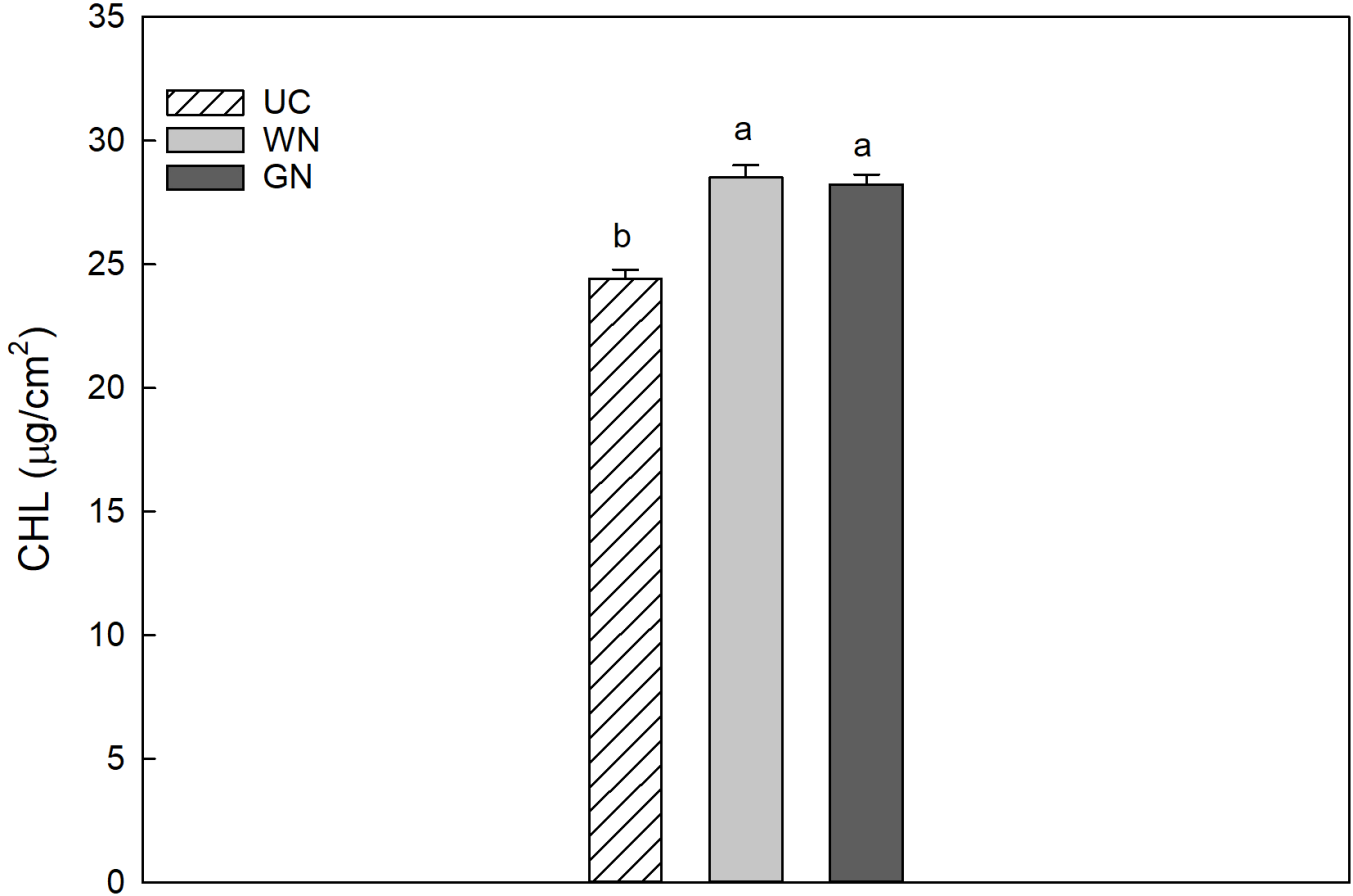
Effect of “Treatment” on leaf chlorophyll content (Chl). The data were subjected to two-way ANOVA (Tukey’s test, p-value <0.05) and different letters indicate a statistically significant difference. ± SE is shown with the bars.

**Figure 6:**
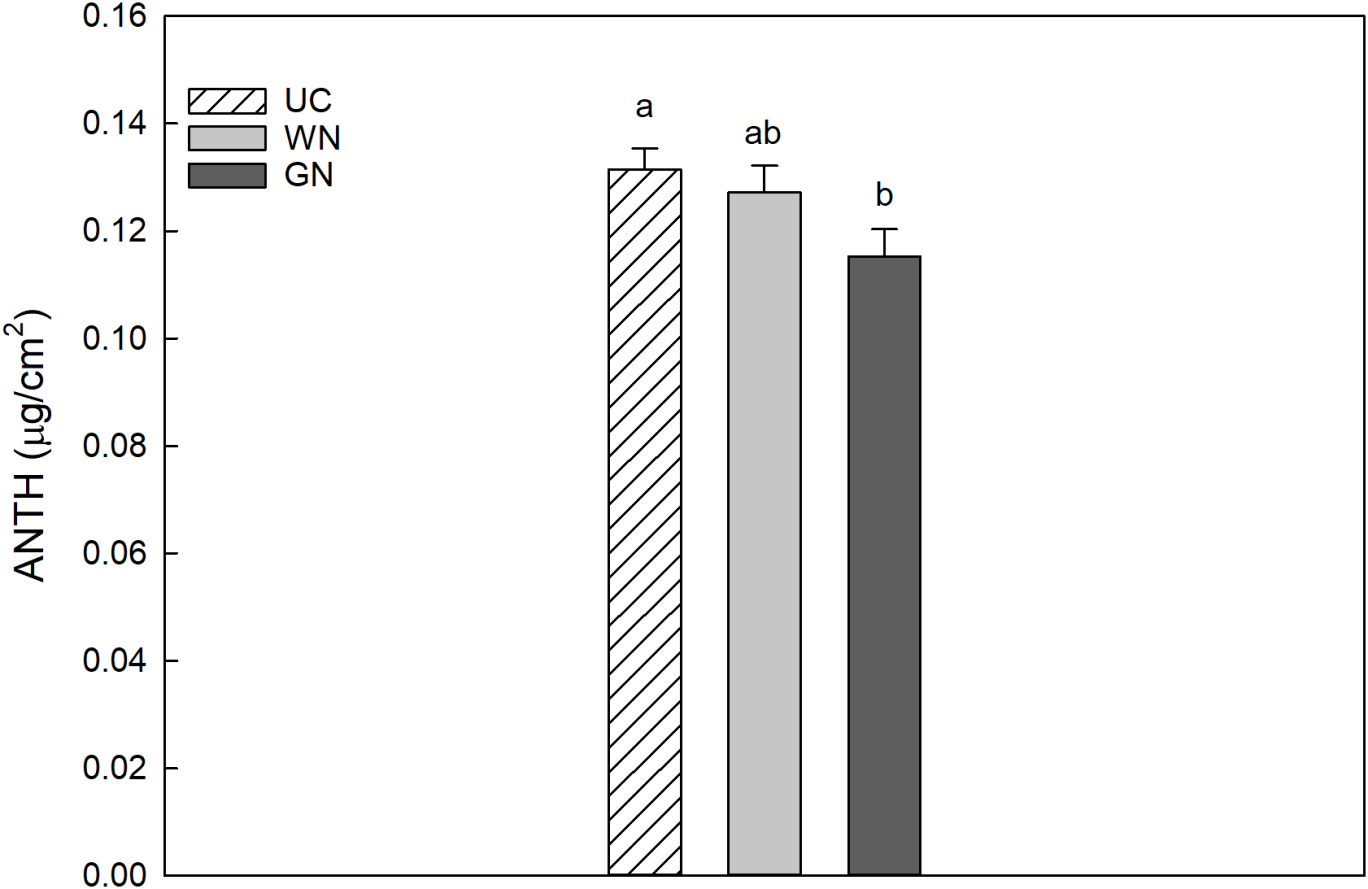
Effect of “Treatment” on leaf anthocyanins content (Anth). The data were subjected to two-way ANOVA (Tukey’s test, p-value <0.05) and different letters indicate a statistically significant difference. ± SE is shown with the bars.

The results for CHL indicated that the average chlorophyll value for WN was 28.53 µg/cm^2^, slightly higher than that for GN (28.24 µg/cm^2^); however, both shaded theses manifested significantly higher values than UC (24.42 µg/cm^2^) (**Figure 5**). The data obtained for leaf chlorophyll content in 2019 and 2020 were 27.80 and 26.33 µg/cm^2^, respectively. This represents a significant difference between the two years, amounting to a decrease of about 5.3% in CHL from 2019 to 2020. The results showed that UC had the highest mean anthocyanin value (0.1314 µg/cm^2^) followed by WN (0.1272 µg/cm^2^), while GN had the lowest mean value (0.1153 µg/cm^2^) (**Figure 6**). The differences in mean anthocyanin values observed among the three theses were statistically significant, with UC having significantly higher values than WN and GN.

**Figure 7:**
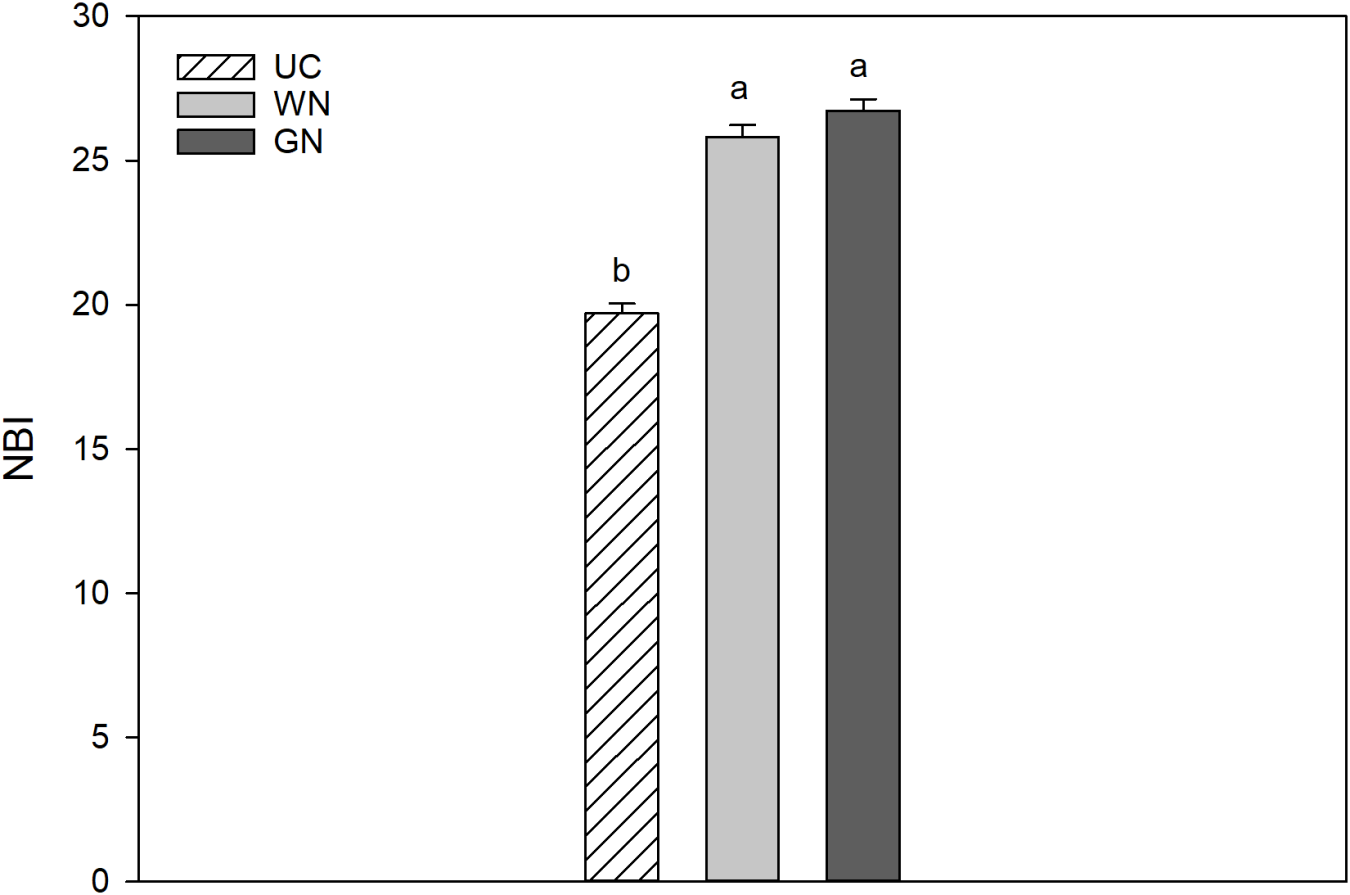
Effect of “Treatment” on Nitrogen Balance Index (NBI). The data were subjected to two-way ANOVA (Tukey’s test, p-value <0.05) and different letters indicate a statistically significant difference. ± SE is shown with the bars.

The ratio of chlorophyll to flavonoids defines the nitrogen balance index (NBI) and thus provides information about the nitrogen status of the plant. Shaded plants showed higher NBI than UC, with NBI index increases of 35.7% (WN) and 31.1% (GN) (**Figure 7**).

### 3.4 Grape yield, and fruit quality

#### 3.4.1 Yield component

The “year” factor was significant for all production attributes analyzed (number of bunches per shoot; berry weight; bunch weight; yield per vine) the same cannot be said for the “treatment” factor, as no difference was found only for the number of bunches per shoot (p = 0.928). The “YxT” interaction was never significant.

**Figure 8:**
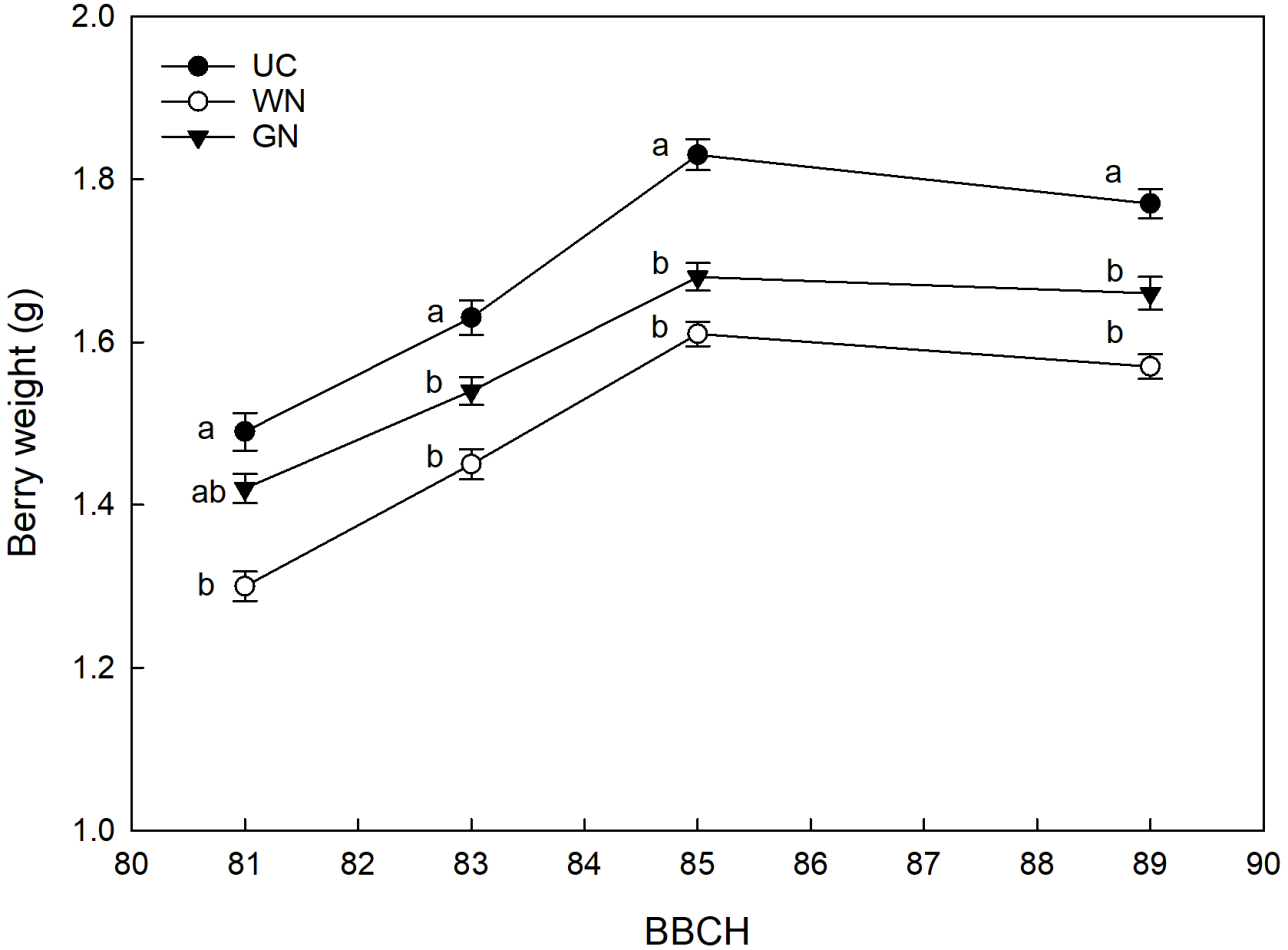
Effect of “Treatment” on average berry weight. The data were subjected to two-way ANOVA (Tukey’s test, p-value <0.05) and different letters indicate a statistically significant difference. ± SE is shown with the bars.

The WN and GN treatments showed significantly lower yield parameters compared to UC, for the four characteristics analyzed. The average berry weight showed an effect of the shading factor from the beginning of ripening until harvest (**Figure 8**). Results for the last sampling date showed that both WN and GN had significantly lower average berry weight values than UC. Specifically, WN had an average berry weight of, 21.5%lower than the average UC value (1.77 g), and GN had an average berry weight of, 14.7% lower. A comparison of the two shaded theses, on the other hand, showed that WN had a berry weight 7.3% lower than GN. This trend was observed by data on average bunch weight. Both, WN and GN had significantly lower average bunch weight values than UC. Specifically, WN had an average bunch weight 29.0% lower than the average UC value, and GN had an average bunch weight 21.1% lower than the average UC value of 0.168 kg (**Table 5**).

**Table 5:** Quantitative parameters of the untreated control and shade treatments measured at harvest (BBCH 89) related to “treatment”.

Treatment	BCW (Kg)		YV (Kg)	
UC	0.168 ± 0.009	a	2.174 ± 0.161	a
WN	0.128 ± 0.007	b	1.629 ± 0.128	b
GN	0.144 ± 0.010	b	1.900 ± 0.133	b

BCW, Bunch weight; YV, yield per vine. Different letters within a column represent significant differences (Tukey’s test, p-value <0.05), n.s. = not significant. Values are reported as means ± SE.

These results are confirmed by the yield values per vine; in particular, the results showed that both WN and GN had significantly lower average yield values per vine than UC. Specifically, WN had an average yield per vine 26.1% lower than the average UC value, while GN had an average yield per vine 17.3% lower than the average UC value of 2.174 kg (**Table 5**).

#### 3.4.2 Berry quality

**Figure 9:**
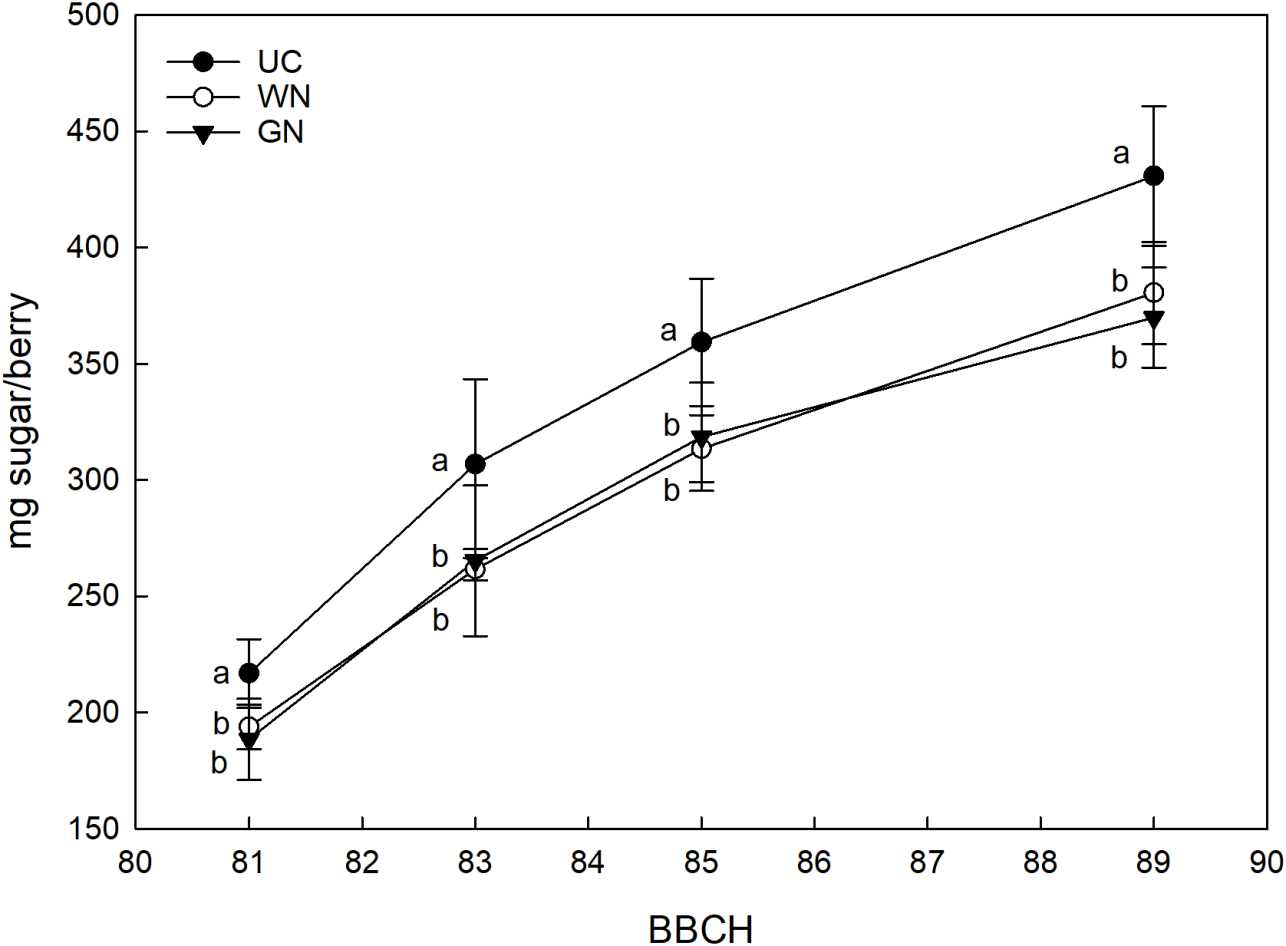
Effect of “Treatment” on sugar per berry (mg). The data were subjected to two-way ANOVA (Tukey’s test, p-value <0.05) and different letters indicate a statistically significant difference. ± SE is shown with the bars.

The ANOVA test revealed that the sugar load expressed in mg sugar per berry was significantly different among treatments. As shown in **Figure 9**, the sugar load increased gradually as the ripening period progressed and reached a plateau phase around mid-August-early September. Already at the beginning of ripening, UC manifested higher mg sugar values of about 11% compared to WN and 15% compared to GN. This trend was observed throughout the period considered until harvest.

The TSS expressed in °Brix was not significantly different between treatments.

**Figure 10:**
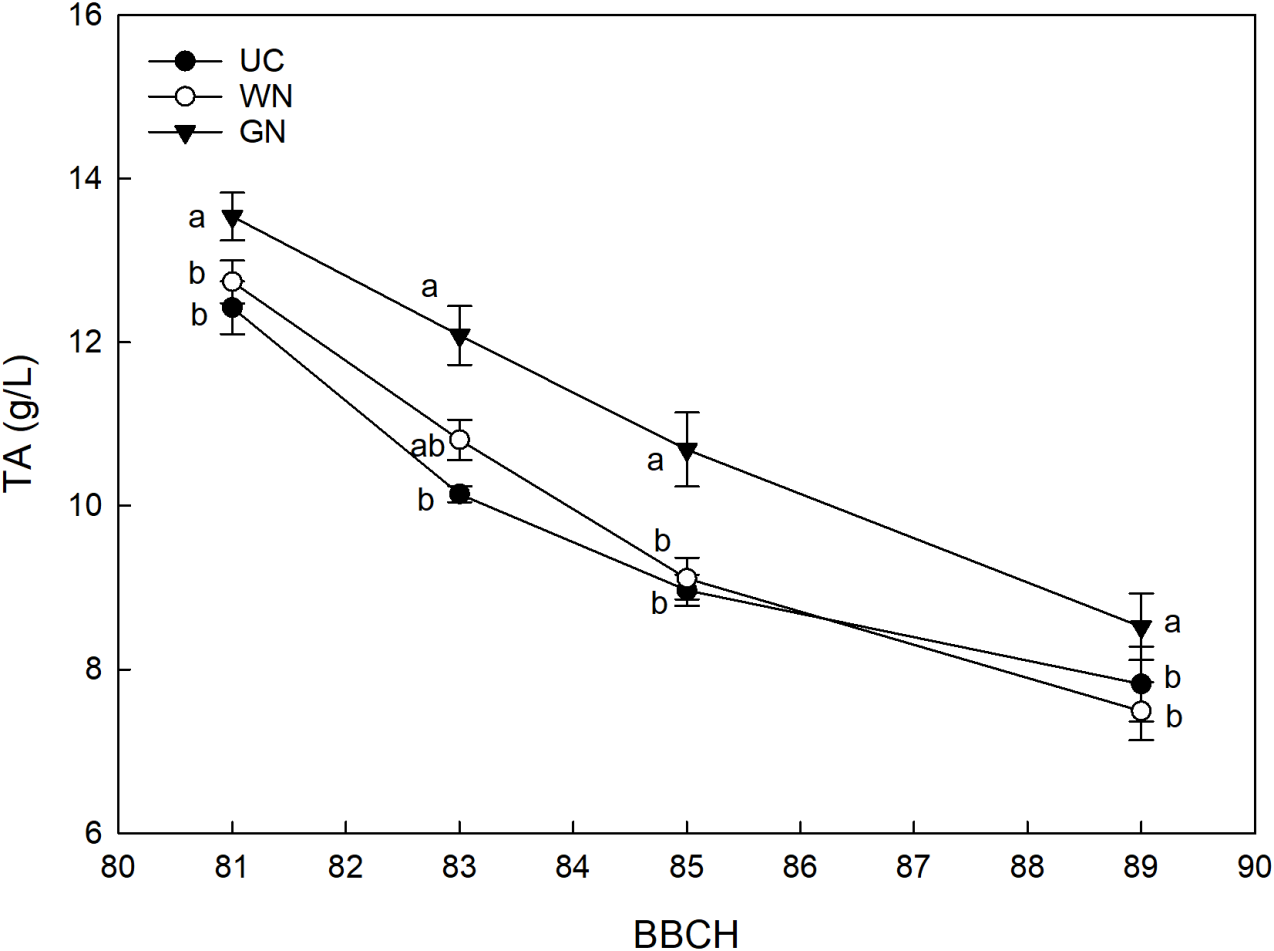
Effect of “Treatment” on TA (g/L). The data were subjected to two-way ANOVA (Tukey’s test, p-value <0.05) and different letters indicate a statistically significant difference. ± SE is shown with the bars.

**Figure 11:**
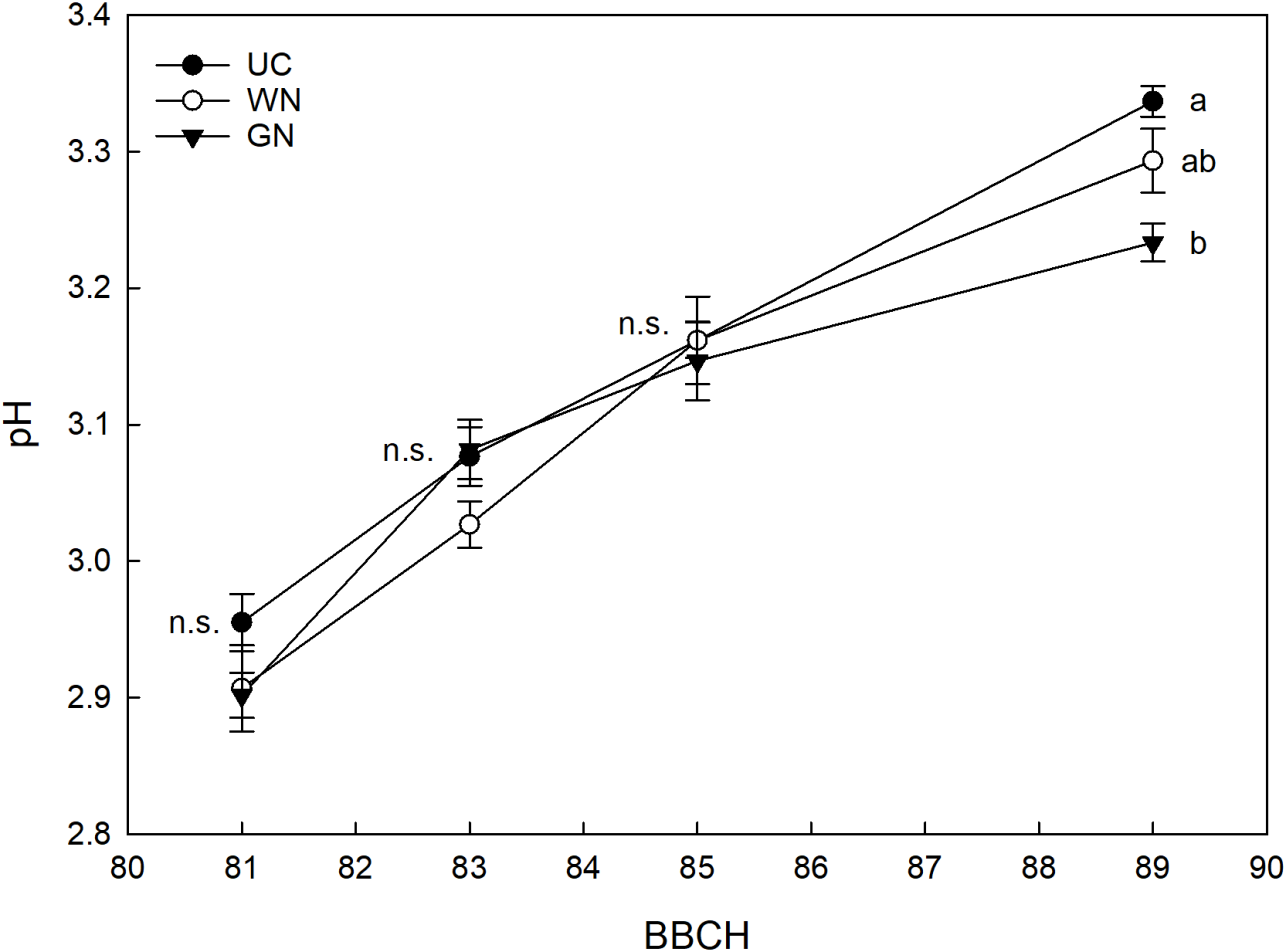
Effect of “Treatment” on pH. The data were subjected to two-way ANOVA (Tukey’s test, p-value <0.05) and different letters indicate a statistically significant difference. ± SE is shown with the bars.

Analysis of grape titratable acidity showed a treatment effect. The most important differences found were in GN, which resulted in an average increase in titratable acidity at harvest of about 9% compared with UC and almost 20% compared with WN (**Figure 10**). **Figure 11** shows the effect of the “treatment” factor on pH. Although until BBCH 85 ST and UC manifested quite similar pH values, significant differences are evident from the data referring to the last sampling date (BBCH89) when the pH level was higher in the UC (3.34) than in the G (3.23), while the WN (3.29) has intermediate values, similar to what it was observed in terms of titratable acidity.

## 4 Discussion

To evaluate the impact of shading on vegetative and productive characteristics, Nero d’Avola vines were shaded with two types of nets that varied in color and shade factor to prevent interception of photosynthetically active radiation (PAR). PAR is a flux of energy within the 380-710 nm waveband (McCree, 1981) whose modulation through canopy management practices modifies the amount of light intercepted by the vines. Previous studies (Kliewer and Lider, 1968; Smart and Sinclair, 1976) indicate a linear correlation between light radiation and temperature; hence, light modification might be a consequence of a significant reduction in temperature. The results of this study indicated a positive correlation between PAR and temperature in GN and UC. However, this response was not as evident in WN. To understand this result, it is important to analyse diffuse reflectance: the transmittance beneath the net is composed of radiation passing through holes in the net and radiation that is scattered downwards from the mesh. The former varies depending on the porosity of the net, while the latter depends on both the mesh structure and the color. Nets with higher solidity and light colors show higher levels of reflectance resulting in higher of diffuse radiation (Al-Helal and Abdel-Ghany, 2010; Al-Helal and Abdel-Ghany, 2011).

This suggests that the microclimate of plants shaded with the white net was predominantly characterized by diffuse radiation, which could explain the lower levels of correlation between temperature and PAR observed in WN. The shading treatments yielded a considerable decrease in both PAR and TMP compared to the unshaded control (UC) in 2020 (**Figure 3B**), yet there were no statistically significant differences between the white net (WN) and UC. The higher temperatures in WN might be attributed to the climatic conditions in 2020 as well as to the specific characteristics of the white net; the higher solidity of the white net results in less incident radiation (with a linear relationship between light radiation and temperature), and simultaneously restricts air circulation, leading to a diminished cooling effect (Whiting and Lang, 2001; Scafidi et al., 2013). This indicates that the response of the white net, in this row orientation, is strongly associated with interannual thermal variations, resulting in a less stable performance compared to the green net.

The lower electromagnetic energy intercepted by the shaded vines resulted in a notable phenological delay of the ST plants compared to UC (**Figure 4**). Thirty days after budburst, when most of the UC shoots were at the visible flowering stage (BBCH53), the GN and WN shoots presented two to three extended leaves (BBCH 12-13). As reported by Scholefield et al. (1978), during the post-harvest period, reserves are recovered; sugars synthesized by the leaves are converted to starch and transferred to woody reserve organs like the roots. In the same way, McArtney and Ferree (1999) report a reduction in the dry weight of shaded grapevine roots; they attribute this result to a lower availability of carbohydrates for the formation of reserves. These considerations highlight the leading role played by the photosynthetic process for the reconstitution of reserves so that a loss of efficiency can seriously compromise the vegetative growth of the following year. In both years, a significant reduction in the development of the main shoots in ST compared to UC was observed. Although plants grown in the shade undergo changes in leaf anatomy and biochemical changes in the photosynthetic apparatus (Boardman, 1977; Kappel and Flore, 1983; McKiernan and Baker, 1991), development in shaded conditions reduces photosynthesis (Givnish, 1988), with obvious repercussions on shoot biomass (Greer and Weston, 2010). In this study, this reduced development of vines in the shade manifested itself on the canes analyzed at harvest and was evidenced by a shorter length and lower number of nodes compared to UC; however, the number of leaves, and consequently also the leaf area per cane and per vine, were significantly higher in ST compared to UC, while no difference was found between WN and GN (**Table 3**). These differences can presumably be attributed to different levels of leaf abscission; indeed, it should be hypothesized that the thermal stress caused by exposure to high PAR levels for a long time, combined with higher leaf temperatures, led to earlier leaf abscission in the UC vines.

In agreement with the observations of Lu et al. (2021) on Cabernet Sauvignon, the shading treatments led to a substantial increase in chlorophyll content (µg/cm^2^) compared to the unshaded control (UC) (**Figure 5**). Other studies (Powles, 1984; Demmig-Adams and Adams, 1992; Takahashi and Murata, 2008) suggest that a prolonged exposure of photosynthetic organs of green plants to high levels of light radiation may result in photoinhibition, This is the result of an apparent photochemical stress condition, which may initially lead to a decrease in photosynthetic performance and eventually lead to the bleaching of pigments (Goh et al., 2012; Guidi et al., 2019). Further investigation of the nutritional status revealed varying accumulations of anthocyanins in the leaves of the three treatments. UC exhibited a significantly higher level of anthocyanins in the upper epidermis.

Previous studies have highlighted the ability of plants to protect themselves from UV radiation damage through the accumulation of phenolic compounds (Burger and Edwards, 1996; Kolb et al., 2003). Their synthesis is enhanced by light (Drumm-Herrel, 1984; Chalker-Scott, 1999) and suppressed by shading or excessive UVB radiation (Camm et al., 1993; Dong et al., 1998).

Our results support the notion that higher levels of this metabolite could be attributed to a condition of photooxidative stress and underscores the potential decrease in photosynthetic capacity of the UC, particularly towards the latter part of the season; this is likely due to the ability of anthocyanins to absorb blue light and reflect red wavelengths (Chalker-Scott, 1999), potentially blocking the photosynthetic activity of the leaves. Also, the concentration of flavonoids which production is promoted by light radiation (Brandt et al., 1995) are significantly higher in UC than in ST treatments. These compounds are believed to be important in UVB protection.

The nitrogen balance index (NBI) is a measure of the ratio of chlorophyll to flavonoids in the leaf epidermis and it can be used to accurately reflect the amount of nitrogen present in a leaf. The NBI is insensitive to plant phenology, meaning that it does not change with the different growth stages of the plant, unlike the two individual indicators. The positive effects of shading were reflected in the NBI, which indicates that the reduction in light intensity due to shading has a beneficial effect on foliar nitrogen content (Cerovic et al., 2015).

In this study, the shading of the vines did not affect the induction and differentiation of inflorescence primordia in any way. This result is by no means taken for granted when considering the fundamental role of light on floral induction. For example, May and Antcliff (1963) demonstrated that a reduction of 30% of full sunlight caused a drastic reduction in the production of the following season. In the same way, Sommer et al. (2002) show that shading caused by particularly expanded canopies resulted in a reduction in the number of inflorescences per node. Therefore, although evaluations for more consecutive years are necessary, this preliminary information seems to suggest that the canopy shading with the nets has no influence on the fruiting of buds. The monitoring of grape berry growth revealed significant differences between the shaded treatments and the control directly exposed; regardless of the season, both GN and WN showed at harvest a reduction in berry weight compared to UC. These results agree with those reported in other studies (Ristic et al., 2007; Koyama and Goto-Yamamoto, 2008) and indicate that berries grown in the absence of light during the initial stages of development show reduced overall growth (Coombe, 1976; Coombe, 1992; Dokoozlian and Kliewer, 1996). However, studies such as that of Martínez-Lüscher et al. (2017) show that there is no one-size-fits-all response; in detail, they report that one of the types of coverings studied determined higher berry weight values in Cabernet Sauvignon compared to the control. In the same way, Cataldo et al. (2021) reported a higher average berry weight for one of the two shaded treatments; Scafidi et al. (2013) reported higher thermal stress values for exposed berries, resulting in a reduction in their average weight. Cases like these, therefore, suggest that direct exposure to sunlight greatly affects berry temperature causing dehydration phenomena that have a strong impact on the final volume of the berries (Greer and Weston, 2010; Carlomagno et al., 2018). As reported by other authors, shading has caused a delay in maturity. The fruits grown under low light conditions showed a lower sugar content, expressed in mg per berry, compared to the ones directly exposed. However, the same variable expressed in Brix reached a similar final value among the three treatments. This last result could be the consequence of the evident lower weight of the berry found in ST, which is likely to have caused a higher concentration of sugars. A clear effect of shading on the total soluble solids content was also reported for Cabernet Sauvignon (Archer and Strauss, 1989), for which single cover has reduced TSSs by almost 2° Brix compared to the control. In a study conducted in Montpellier, France, Syrah vines shaded from fruit set to harvest showed that the sugar content of grapes was lower as the intensity of the canopy shading increased (Bureau et al., 2000). A strong effect of shading is also reported on Syrah vines grown in Adelaide, Australia; specifically, Caravia et al. (2016) report that the exclusion of 62% of the total radiation with nets placed above the canopy on average caused a reduction of 1.5° Brix compared to control. The general indication is that these results may be the reflection of the lower exposure of the leaves to the sun, which causes a reduced assimilation of photosynthetics for the maturing bunches (Downey et al., 2004). The already mentioned delay in ripening for shaded grapes is confirmed by the increase in titratable acidity (g/L); the pH of the berries was found to be inversely correlated to the acidity (Spayd et al., 2002). These results are confirmed by previous studies (Scafidi et al., 2013; Martínez-Lüscher et al., 2017) and show that the titratable acidity values reflect the berry temperature (Hale and Buttrose, 1974). In detail, GN has shown the constant ability to reduce the respiratory processes of organic acids with the consequent increase in acidity (Buttrose et al., 1971). The same cannot be said for WN; the latter showed an unstable effect, being more subject to the environmental conditions that characterized each growing season. This statement takes on greater value when considering the technical characteristics of WN; as mentioned before, in fact, the white color determines an increase in the effects of diffuse radiation.

## 5 Conclusion

This study shows that the canopy coverage with nets caused partial changes of the microclimate which reduced the thermal and luminous stress of the canopy and a slowdown in the ripening process. These effects are related to the current problems of climate change. Moreover, the shade, although acting negatively on the number of bunches produced, has proven to be an effective tool for obtaining smaller berries, grapes with lower sugar content and less degradation of the acid structure. The net technical characteristics seem to have an important impact on the amount of light and air circulation that the plants receive, which can therefore have a strong effect on vegetative production and microclimate. Is presumable that the white net has greater reflectance of diffuse radiation than the green nets, resulting in lower levels of PAR. This has caused a lower correlation between temperature and PAR in the white net. Also, the white net had a greater impact on leaf abscission, berry weight, and total soluble solids. The green net, on the other hand, was better at reducing the respiratory processes of organic acids and increasing acidity, as well as delaying ripening. These preliminary results show a significant effect of shading level and suggest that the use of nets of different shading factor is a valid option to adapt viticulture to current climate change conditions, however, it will be necessary to carry out further tests aimed at defining the effects of a shading obtained with nets of different colors and mesh texture as well as different values of shading and moments of application of the covering.

## Data availability statement

The raw data supporting the conclusions of this article will be made available by the authors, without undue reservation. 

## Author contributions

DM processed the experimental data, performed the analysis. MR verified the analytical methods. RL supervised the project. SP and MVF performed the measurements. AP conceived and planned the experiments. All authors contributed to the article and approved the submitted version.
